# The Repurposing of Acetylsalicylic Acid as a Photosensitiser to Inactivate the Growth of Cryptococcal Cells

**DOI:** 10.3390/ph14050404

**Published:** 2021-04-23

**Authors:** Adepemi O. Ogundeji, Nozethu Mjokane, Olufemi S. Folorunso, Carolina H. Pohl, Martin M. Nyaga, Olihile M. Sebolai

**Affiliations:** 1 Department of Microbiology and Biochemistry, University of the Free State, Bloemfontein 9301, South Africa; ogundejiao@ufs.ac.za (A.O.O.); nmjokane@gmail.com (N.M.); foxyphemmzy@gmail.com (O.S.F.); pohlch@ufs.ac.za (C.H.P.); 2 Next Generation Sequencing Unit and Division of Virology, University of the Free State, Bloemfontein 9301, South Africa; nyagamm@ufs.ac.za

**Keywords:** acetylsalicylic acid (ASA, aspirin), capsule, *CAP64*, *Cryptococcus*, membrane potential (ΔΨM), photodynamic treatment, photosensitiser, ultrastructure

## Abstract

Photodynamic treatment (PDT) is often successful when used against aerobic microbes, given their natural susceptibility to oxidative damage. To this end, the current study aimed to explore the photodynamic action of acetylsalicylic acid (ASA; aspirin, which is commonly used to treat non-infectious ailments), when administered to respiring cryptococcal cells. The treatment of cryptococcal cells, i.e., exposure to 0.5 or 1 mM of ASA in the presence of ultraviolet light (UVL) for 10 min, resulted in a significant (*p* < 0.05) reduction in the growth of tested cells when compared to non-treated (non-Rx) cells, i.e., no ASA and no UVL. The treated cells were also characterised by diseased mitochondria, which is crucial for the survival of respiring cells, as observed by a significant (*p* < 0.05) loss of mitochondrial membrane potential (ΔΨM) and significant (*p* < 0.05) accumulation of reactive oxygen species (ROS) when compared to non-Rx cells. Moreover, the photolytic products of acetylsalicylic acid altered the ultrastructural appearance of treated cells as well as limited the expression levels of the capsular-associated gene, *CAP64*, when compared to non-Rx cells. The results of the study highlight the potential use of ASA as a photosensitiser that is effective for controlling the growth of cryptococcal cells. Potentially, this treatment can also be used as an adjuvant, to complement and support the usage of current anti-microbial agents.

## 1. Introduction

Cryptococcosis caused by the fungus *Cryptococcus* (*C*.) *neoformans*, is one of the most common opportunistic infections with a significant mortality rate among AIDS patients, more especially in resource-limited countries such as in sub-Saharan Africa [1,2,3,4,5,6,7]. In these vulnerable subjects, i.e., with a depleted cell-mediated immune response, fungal cells can disseminate to the brain. There, the cells impair the ability of the brain to reabsorb the cerebrospinal fluid, leading to a build-up within the skull as well as causing meningitis [8,9]. Such a patient can then present with a debilitating headache, coma, or even die. It is reported that 223,100 cases of cryptococcal meningitis occur annually, of which 73% are diagnosed in sub-Saharan Africa [2,3,4,10]. Furthermore, the mortality rate of this disease in sub-Saharan Africa is 75% [2,3,4,10].

This fungus can also cause cutaneous infections, which are not as prevalent as cryptococcal lung or central nervous system infections [11,12]. The manifestation of cryptococcal skin infections, i.e., draining sinuses, acneiform lesions, among others, is typically an indication of systemic infection and not direct inoculation [11,12]. In the case of the former, cryptococcal cells may be transported via a haematogenous route or invade circulating macrophages and, in a manner akin to the Trojan horse, reach the skin [8,9]. These skin infections are reported to occur in 10–15% of patients with an invasive cryptococcal infection [11].

To control cryptococcal infections, three drugs, namely, fluconazole, amphotericin b and flucytosine, have been recommended for management purposes [13,14]. However, in South Africa, only fluconazole and amphotericin b are routinely used in public health institutions [7,15,16,17]. To compound this, the clinical application of these two drugs is often limited. For example, fluconazole is usually used as a first-line treatment. Although this drug has been shown to easily cross the blood–brain barrier, it is, however, also reported to have relatively poor fungal clearance, even when administered at high doses [5,18]. On the other hand, amphotericin b is a better choice due to its effectiveness but has huge toxicity, and its administration is usually intravenous [18,19]. Due to these shortcomings, these medicines are thus often associated with clinical failure [20,21,22]. 

Given the medical importance of cryptococcal infections, several scholars have looked at other strategies, such as the repurposing of non-traditional antifungals in combating this deadly pathogen [21,23,24,25]. Thus, the current study aims to repurpose acetylsalicylic acid (ASA; aspirin) as a photosensitiser. ASA is an old drug that is recommended to treat pain and inflammation [26,27]. It is, therefore, not surprising that ASA is on the World Health Organization’s List of Essential Medicines [28]. Importantly, the wholesale cost of ASA in the developing world is estimated to be USD 0.002 to USD 0.025, as of 2014 [29]. 

A photosensitiser is a compound which, when excited by an appropriate light source, could generate harmful radicals [30]. ASA, like other aromatic carboxylic acids, has intense absorption bands in the UV spectral range [31]. However, the photochemical properties of molecules such as salicylates (SAs) and their derivatives are said to be poorly understood [31].

**Figure 1:**
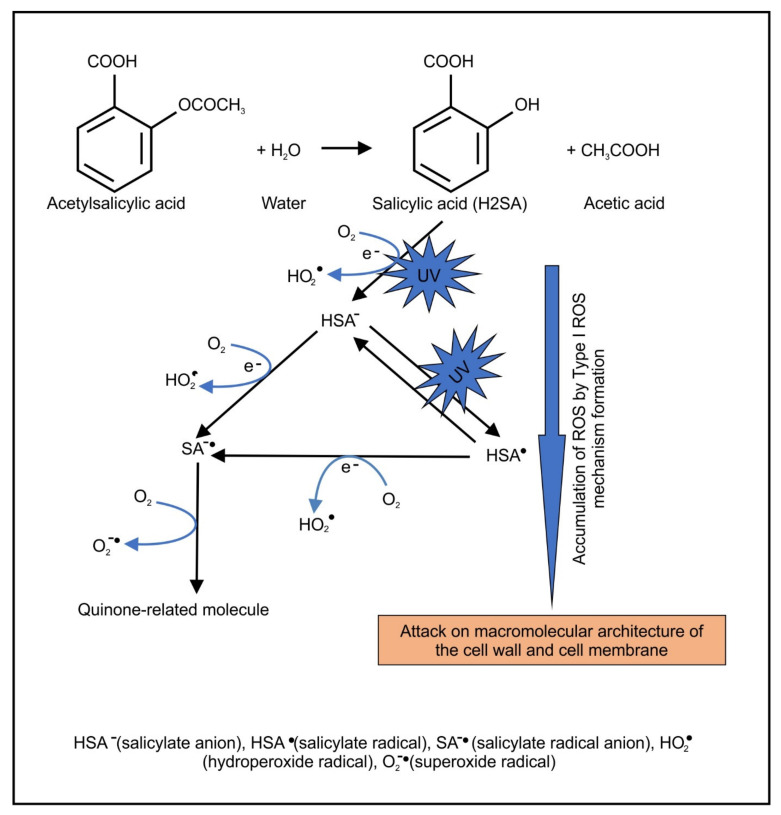
Proposed mechanism of UV-induced photosensitizer and accumulation of ROS via Type I mechanism.

SA is the active compound of ASA, and it is responsible for its biological properties. Through using optical spectroscopy, fluorescence spectroscopy, and nanosecond laser flash photolysis, Pozdnyakov et al. [31] showed that excitation of SA could yield a salicylate anion triplet state (HSA^−^), HSA· radical and hydrated electron (e_eq_^−^), wherein the last two species are possibly generated due to two-photon processes [32,33]. These radicals can then react with molecular oxygen to generate reactive oxygen species (ROS) typifying a Type I reaction (Figure 1). 

The ability of ASA to generate ROS was also shown in a study by Ogundeji et al. (2016) [25]. In the study, these authors showed that ASA engaged a signalling programme that involved the participation of a high osmolarity glycerol (HOG) pathway. The activation of this pathway suggested that ASA-induced ROS-based oxidative stress was sufficient to kill cryptococcal cells [25]. In their study, Norman et al. [34] also showed that SA could uncouple the electron transport chain. In turn, the latter impairs the oxygen (as a final electron acceptor) from receiving a full complement of electrons, thus leading to the production of ROS [34].

In the current study, ASA was used at concentrations (0.5 and 1 mM) that are within the recommended threshold [35]. Based on the physiology of cryptococcal cells, i.e., they are non-fermentative [36], it was hypothesised that this respiring organism would be susceptible to stress induced by harmful radicals, as previously shown after treatment with ASA [25].

## 2. Results

### 2.1. Cryptococcal Cells Are Susceptible to the Photodynamic Action of ASA

**Figure 2:**
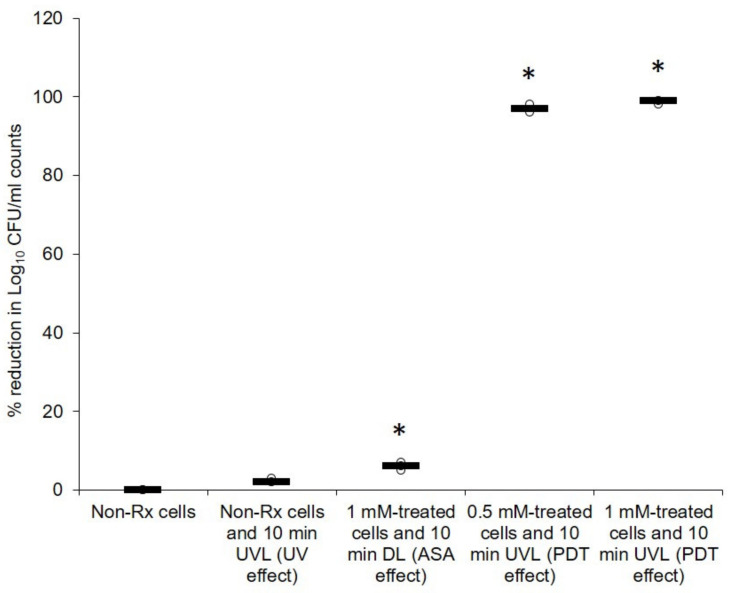
Effect of PDT with ASA on the survival of *C. neoformans* LMPE 046. Non-Rx, non-treated cells; UVL, ultraviolet light; DL, dark light; CFU, colony-forming unit. Data points were obtained from three biological replicates for each defined experimental condition. * Significantly different from non-Rx cells at *p* < 0.05.

Figure 2 summarises the effect of PDT with ASA on the survival of the tested cryptococcal cells. As expected, the negative controls, i.e., 1 mM of ASA alone (with no cells), and 1 mM of ASA and 10 min UVL (with no cells), and no CFUs were observed on the agar plates after incubation (data not shown). When used alone, neither ASA nor UVL was able to yield a reduction in CFU counts that were above 10% when compared to non-treated (non-Rx) cells counts, i.e., no ASA and no UVL. However, when used in combination, a compounded effect was observed. Moreover, there was a significant (*p* < 0.05) reduction (equal to and above 97%) in CFU counts when PDT was applied (0.5 mM and 10 min UVL or 1 mM and 10 min UVL), when compared to non-Rx cells’ counts. A similar response profile was obtained when the following cryptococcal strains were exposed to similar conditions *viz*. *C. neoformans* H99, *C. gattii* LMPE 052 and *C. gattii* R265 (Figure S1).

### 2.2. Light-Activated ASA Kills Cryptococcal Cells via the Production of Harmful Radical Species That Target Cell Walls

**Figure 3:**
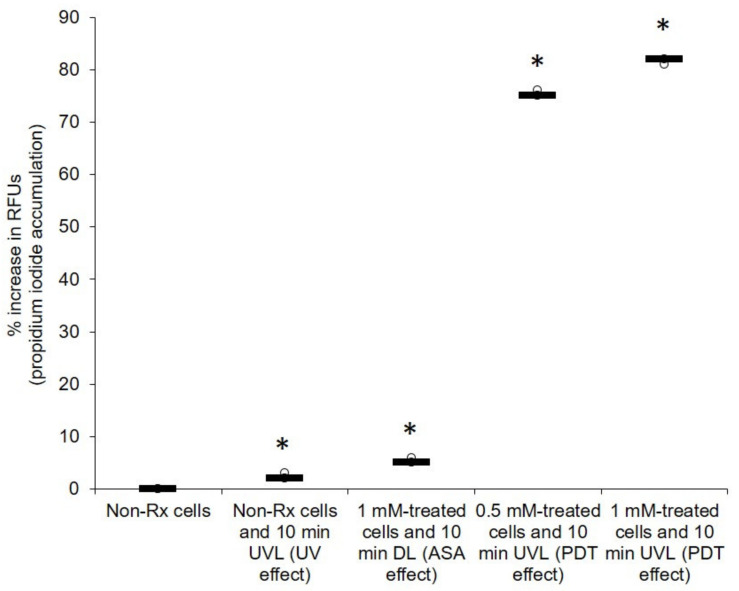
PDT action of ASA showing that cells of *C. neoformans* LMPE 046 lost their selective permeability. The background signal of negative controls was used for normalisation. There was a normality of data distribution. Non-Rx, non-treated cells; UVL, ultraviolet light; DL, dark light; RFUs, relative fluorescence units. Data points were obtained from three biological replicates for each defined experimental condition. * Significantly different from non-Rx cells at *p* < 0.05.

Given that the photolytic products of ASA may be harmful, it was sought to determine if these products may also target the cytoplasmic membrane and ultrastructural features of cryptococcal cells. It was, therefore, not surprising when studying the effects of PDT on the integrity of the cell membrane (using the PI exclusion assay) to note that all treated cells (0.5 mM or 1 mM) following exposure to UVL (10 min) had significantly (*p* < 0.05) accumulated the PI stain in the cytoplasm, while the corresponding non-RX cells did not accumulate the stain (Figure 3). The above findings suggested that the membranes of the treated cells had lost their selective permeability. 

**Figure 4:**
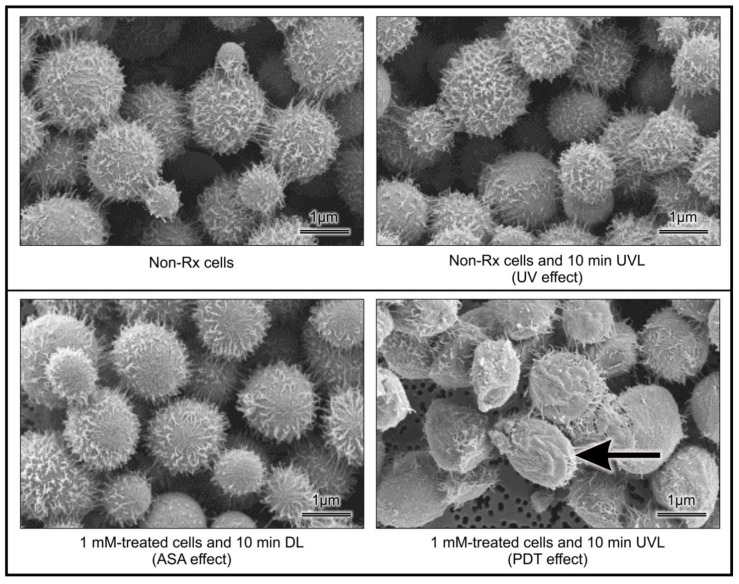
SEM images showing the morphological differences of cryptococcal cells’ ultrastructure after being handled in designed experimental groups. The arrow points at cells which exemplify the effects of PDT, i.e., a cell that appears wrinkled. The images were taken after studying *C. neoformans* LMPE 046 cells.

The captured micrographs (representing cells obtained from the different experimental conditions) were collated into Figure 4, to aid in making deductions regarding the effect of PDT. The figure showed that the harmful ASA photolytic products may have targeted the cell walls of cells that were exposed to the combined effect of ASA and UVL. This assertion is based on comparing the morphological differences of cells between the different experimental conditions. Furthermore, *C. neoformans* LMPE 046’s non-Rx cells appeared to be whole and covered with web-like extracellular matrixes (possibly the capsule) on their cell wall surfaces. Exposure of cells to ASA treatment and separately to UVL treatment did not effect a change in the appearance of these cells when compared to non-Rx cells. However, the exposure of cells to PDT led to an ultrastructural change, because cells were observed to have collapsed cell walls, and some appeared to have less of the web-like extracellular matrixes. 

**Figure 5:**
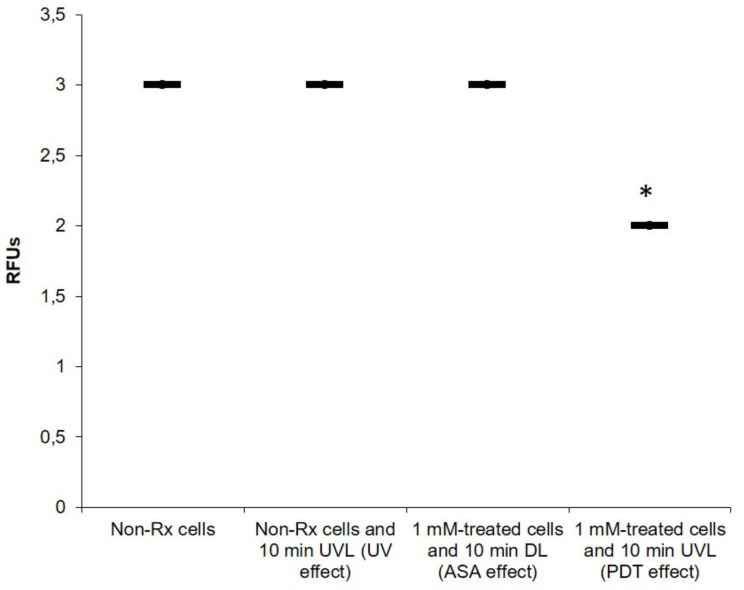
The effect of different experimental conditions on the expression of the *CAP64* gene in *C. neoformans* LMPE 046. Non-Rx, non-treated cells; UVL, ultraviolet light; DL, dark light; RFUs, relative fluorescence units. Data points were obtained from three biological replicates for each defined experimental condition. * Significantly different from non-Rx cells at *p* < 0.05.

The morphological changes may be incidental due to sample preparation; assessment of the impact of PDT on the expression of a capsular gene responsible for capsule formation was also sought. The effects of the different experimental conditions on the expression of the *CAP64* gene were summarised in Figure 5. When comparing the non-Rx cell data, it was noted that PDT had an effect on the capsule of *C. neoformans* at a genomic level; consequently, the expression of *CAP64* was significantly reduced (*p* < 0.05), as shown in Figure 5. Low expression of a Cap gene, particularly *CAP64* in this case, may result in an acapsular phenotype, as previously documented [37,38]. On the other hand, there was no significant difference (*p* > 0.05) in the expression of the *CAP64* gene, when the non-Rx cell data were compared to the ASA effect and separately to UVL effect. The implication of the above is that cryptococcal cells exposed to PDT with ASA would be more vulnerable to the action of antimicrobial drugs. It is documented elsewhere that the capsule can impair the transport and uptake of antifungals such as amphotericin B into the cell cytoplasm [39]. Moreover, in the context of an immune response, lack of or a compromised capsule implies that cells would be easily targeted by host immune molecules and cells [40]. It is important to note that our work is preliminary to draw concrete conclusions on the effects of PDT treatment on the cryptococcal capsule. To this end, supporting evidence related to the downregulation of additional cell structures or cell wall genes is required.

### 2.3. The Photodynamic Action of ASA Impairs Cryptococcal Mitochondrial Function 

**Figure 6:**
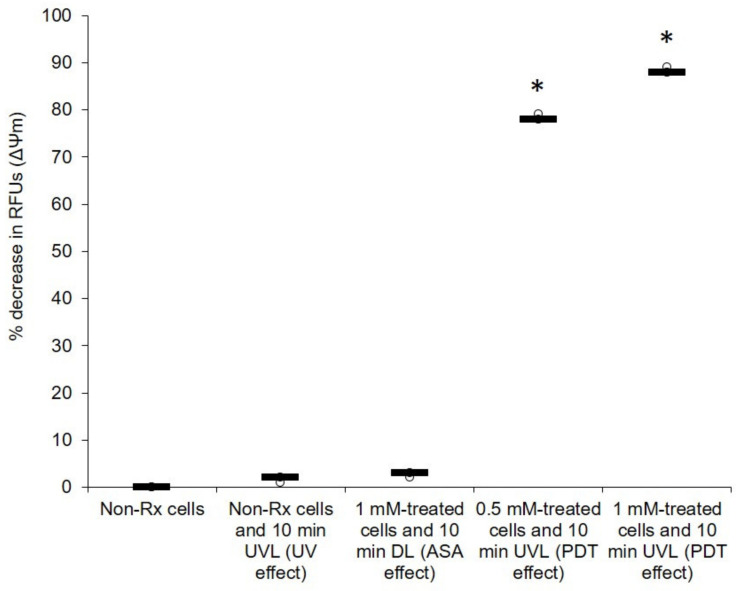
PDT action of ASA impairs the energising of mitochondrial membranes of *C. neoformans* LMPE 046 cells. The background signal of negative controls was used for normalisation. There was a normality of data distribution. Non-Rx, non-treated cells; UVL, ultraviolet light; DL, dark light; RFUs, relative fluorescence units. Data points were obtained from three biological replicates for each defined experimental condition. * Significantly different from non-Rx cells at *p* < 0.05.

**Figure 7:**
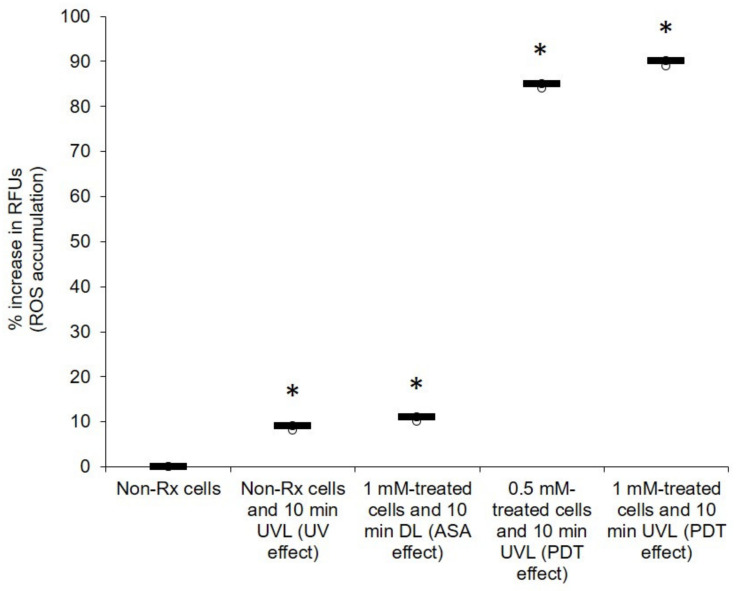
PDT action of ASA impairs the mitochondrial electron transport chain leading to excessive ROS production in *C. neoformans* LMPE 046. The background signal of negative controls was used for normalisation. There was a normality of data distribution. Non-Rx, non-treated cells; UVL, ultraviolet light; DL, dark light; RFUs, relative fluorescence units. Data points were obtained from three biological replicates for each defined experimental condition. * Significantly different from non-Rx cells at *p* < 0.05.

Oxygen is an essential element for obligate, respiring microbes [41]. It is required for their survival because it is used in oxidative phosphorylation, a process that leads to cellular energy production [42]. Therefore, any impairment to the above process that results in oxygen not receiving a full complement of electrons to reduce it leads to the production of harmful oxygen radicals [43]. Herein, we show that the photosensitising effect of ASA seems to disrupt the normal functioning of mitochondria (Figure 6). When comparing the membrane potential (ΔΨM) of all the tested *C. neoformans* strains (treated with 0.5 mM or 1 mM of ASA in the presence of UVL for 10 min) to that of their corresponding non-Rx cells, it was noted that the effect of ASA was enhanced in the presence of UVL; these cells (exposed to the combined effect of ASA and UVL) had significantly (*p* < 0.05) lost their membrane potential (ΔΨM). ROS accumulation was also measured in all treated cells (0.5 mM or 1 mM in the presence of UVL (10 min)) as well in their corresponding non-Rx cells (Figure 7). As expected, the combined effect of ASA and UVL resulted in cells significantly accumulating more than 80% (*p* < 0.05) of ROS when compared to their respective non-Rx cells. Therefore, this turn of events in the form of a collapsing mitochondrion starves cells of potential energy to support important cellular processes such as growth [44].

### 2.4. The PDT Action of ASA Does Not Adversely Affect the Health of Macrophages

**Figure 8:**
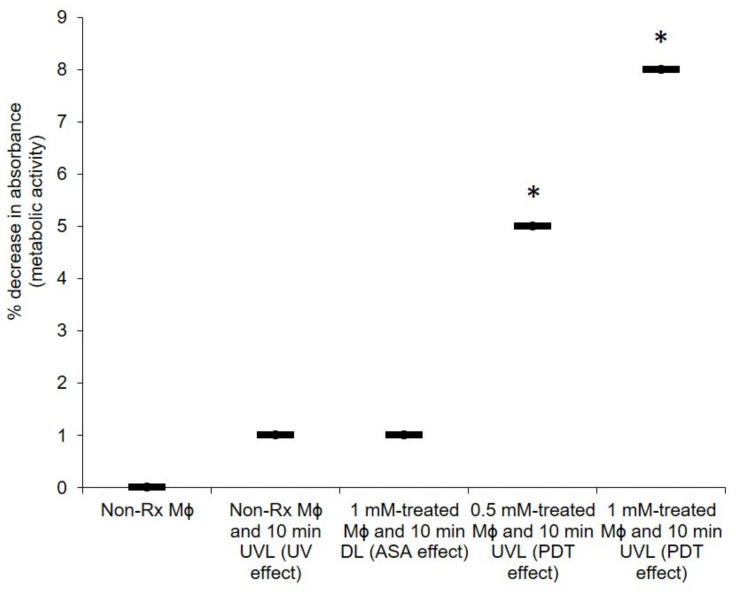
ASA is a suitable photosensitiser to apply against macrophages. The background signal of negative controls was used for normalisation. There was a normality of data distribution. Non-Rx, non-treated cells; UVL, ultraviolet light; DL, dark light; and Mɸ, macrophages. Data points were obtained from three biological replicates for each defined experimental condition. * Significantly different from non-Rx cells at *p* < 0.05.

Crucial to the pathogenesis of *C. neoformans* is the ability of cells to manipulate macrophages in a Trojan horse-like manner for dissemination purposes [45]. Thus, for ASA to be considered as an ideal photosensitiser, it should also not be detrimental to the host cells. Here, it was determined that PDT with ASA did not kill more than 10% of the macrophage population (0.5 mM-treated cells and 10 min UVL exposure or 1 mM-treated cells and 10 min UVL exposure) when compared to the non-Rx macrophage population (i.e., no ASA and no UVL) as seen in Figure 8. Moreover, after a 10 min exposure period, only 5% of the population was killed using 0.5 mM, while 1 mM killed 8%. Importantly, in all these cases, the application of ASA did not effect a lethal dosage of 50 (LD_50_), wherein 50% of the tested macrophage population would have been killed.

## 3. Discussion

Antimicrobial resistance has become a major contributor to mortality, and this has necessitated the quest to find alternative ways of controlling infections. One avenue that was considered in this study was the susceptibility of the fungal pathogen *C. neoformans* to PDT with ASA. We showed that when either ASA or UVL is used alone, they are unable to kill a significant number of cryptococcal cells. This speaks to the principle of PDT, wherein a photosensitiser is only effective when activated by a specific light. 

The idea that *Cryptococcus* would be susceptible to PDT is not surprising, based on the theory by Kock and co-workers that organisms with a strict aerobic metabolism (such as *Cryptococcus*) are more susceptible to death induced by oxidative damage as a result of impairment to mitochondrial respiration than those that can also produce energy through an alternative anaerobic glycolytic fermentative pathway in which mitochondria are not involved [46]. The above theory was shown to be true when tested on additional strains belonging to the *C. neoformans* species complex (Figure S1).

While this technology has mainly been used in the treatment of cancers, there is a body of work that has shown a successful application against mycotic agents. To illustrate this point, in 2010, Mang and co-workers demonstrated the fungicidal effect of PDT against *Candida* species [47]. From their observations, 25 µg/mL of porfimer sodium killed more than 90% of *Candida* strains following illumination [45]. This is important because these fungi are often problematic to manage in AIDS patients, because they are resistant to fluconazole and amphotericin B [48,49]. There may be hope on the horizon; a new study by Nagy et al. [50] recently demonstrated the in vitro antimicrobial success of using 1-amino-5-isocyanonaphthalene [ICAN] against fungal pathogens [50]. Based on the aromatic fluorophore nature of ICAN, it would be prudent to also assess its photosensitising quality. PDT has also been shown to be effective against some *Cryptococcus* species. In their study, Fuchs et al. [51] demonstrated the susceptibility of *C. neoformans* towards a polycationic conjugate of polyethyleneimine and chlorin (as the photosensitiser). In 2011, Soares et al. [52] also reported the efficacy of PDT against a number of *C. gattii* isolates with different susceptibility profiles, suggesting that PDT could be an alternative strategy to inhibit cryptococcal cells.

**Figure 9:**
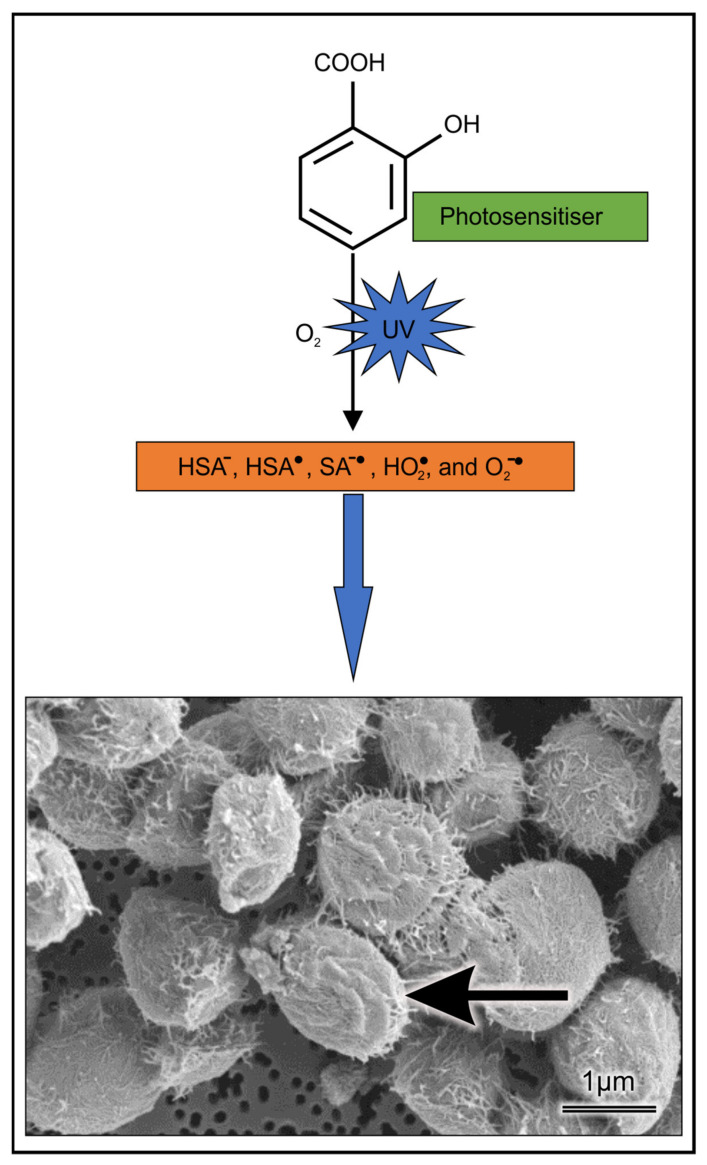
Deleterious effect of accumulated radicals impairs the ultrastructure and integrity of *C. neoformans* LMPE 046 cell walls.

Importantly, we further showed that harmful radical species possibly killed cryptococcal cells. Based on our results, it is conceivable that the generated radicals can then target the cell wall of the cryptococcal cells, as shown in Figure 5 and Figure 6, and in summary, the mode of action is depicted in Figure 9. It important to note that in a biological system (infected host organism), it is possible that SA’s ROS-driven photodynamic effect may be counteracted by SA’s powerful hydroxyl radical-scavenging capacity [53]. To address the latter, animal studies should be considered.

## 4. Materials and Methods

### 4.1. Materials

Yeast extract, malt extract, peptone, glucose, agar (Merck, Johannesburg, South Africa), phosphate buffer solution (PBS) (Sigma-Aldrich, Johannesburg, South Africa), RPMI-1640 medium, foetal bovine serum (Biochrom, Berlin, Germany), penicillin, streptomycin (Sigma-Aldrich, St. Louis, MO, USA), l-glutamine (Sigma-Aldrich, Johannesburg, South Africa), trypan blue stain, sterile disposable 96-well flat-bottom microtitre plate (Greiner Bio-One, Frickenhausen, Germany), acetylsalicylic acid (C_9_H_8_O_4_, MW 180.158 g/mol) (Sigma-Aldrich, Johannesburg, South Africa), absolute ethanol (Merck, South Africa), propidium iodide (PI) (Life Technologies, Carlsbad, CA, USA), sodium-phosphate-buffered 3% glutardialdehyde (Merck, Johannesburg, South Africa), sodium-phosphate-buffered 3% osmium tetroxide (Merck, Johannesburg, South Africa), Beadbug microtube homogeniser (Lasec, Johannesburg, South Africa), 1.0% (*w*/*v*) agarose gel, Tris-acetate-EDTA (TAE) buffer, ethidium bromide (Merck, Johannesburg, South Africa), DNAase (Qiagen, Hilden, Germany), dark reader transilluminator (Clare Chemical Research, Dolores, CO, USA), 5,5′,6,6′-tetrachloro-1,1′,3,3′-tetraethylbenzimidazolylcarbocyanine iodide (JC-1) (Life Technologies, Carlsbad, CA, USA), 2′,7-dichlorofluorescein diacetate (DCFHDA) (Sigma-Aldrich, Johannesburg, South Africa), 2,3-bis (2-methoxy-4-nitro-5-sulfophenyl)-5-[(phenylamino)carbonyl]-2H-tetrazolium hydroxide (XTT; Sigma-Aldrich, Johannesburg, South Africa), 1 mM of menadione (Sigma-Aldrich, Johannesburg, South Africa), germicidal ultraviolet light (UVL) lamp (ESCO, Johannesburg, South Africa), Airstream® Class II Biological Safety Cabinet (ESCO, Johannesburg, South Africa), SEM coating system (Bio-Rad Microscience Division, Johannesburg, South Africa), Shimadzu Superscan SSX 550 SEM (Shimadzu, Tokyo, Japan) and Fluoroskan Ascent FL microplate reader (Thermo Scientific, Waltham, MA, USA).

### 4.2. Cells, Cultivation and Standardisation

The fungal strain *C. neoformans* LMPE 046 was used in this study. The strain was obtained from a patient at the Universitas Academic Hospital, Bloemfontein, South Africa. The strain was grown on yeast–malt-extract (YM) agar (3 mg/mL yeast extract, 3 mg/mL malt extract, 5 mg/mL peptone, 10 mg/mL glucose, 16 mg/mL agar) at 37 °C for 48 h. Before use, 10 mL of phosphate buffer solution (PBS) was used to prepare standardised inocula with a final concentration of between 0.5 × 10^5^ and 2.5 × 10^5^ CFU/mL, according to the protocol of the European Committee on Antimicrobial Susceptibility Testing [54].

The RAW 264.7 macrophage cell line (originally obtained from ATCC) was cultivated in RPMI-1640 medium that was supplemented with 10% foetal bovine serum, 20 U/mL penicillin, 20 mg/mL streptomycin and 2 mM l-glutamine at 37 °C and 5% CO_2_ until 80% confluence was achieved. The viability of the cells was determined to be 90% using a trypan blue stain. This stain is excluded by viable cells and accumulates inside dead cells. Next, macrophages were standardised using a haemocytometer to reach a final cell concentration of 1 × 10^6^ cells/mL in fresh RPMI-1640 media. A 100 µL suspension of macrophages was seeded into wells of a sterile, disposable 96-well flat-bottom microtitre plate and left overnight in a humidified 5% CO_2_ incubator at 37 °C.

### 4.3. UV Radiation

Photosensitiser: Acetylsalicylic acid (C_9_H_8_O_4_, MW 180.158 g/mol) was obtained as a standard powder from Sigma-Aldrich. ASA stock solution was prepared in absolute ethanol to yield a stock solution of 10 mM. The drug was further diluted in RPMI-1640 media in order to reach final concentrations of 0.5 mM and 1 mM. The final amount of ethanol in RPMI-1640 media never exceeded 1%. The UV/Vis absorption of ASA was determined to be 250 nm (data not shown). 

Light source: A germicidal ultraviolet light (UVL) lamp that was fitted inside an Airstream® Class II Biological Safety Cabinet was used as the light source. The lamp is reported to have a nominal power of 30 watts, and thus provided a germicidal UV intensity (irradiance or radiation output) of approximately 125 µW/cm^2^ one metre from the lamp [55]. In the current study, the distance between the lamp and the position of the experimental microtiter plates was 20 cm.

### 4.4. Preparation of Cells for Experimental Assays

A 100 µL suspension of the standardised cryptococcal inoculum (0.5 × 10^5^ and 2.5 × 10^5^ CFU/mL) was added to sterile 96-well flat-bottom microtitre plate wells. Thereafter, 100 µL of the photosensitiser at twice the desired final concentrations was added. A number of experimental conditions were set up at room temperature and these were: (1) non-Rx cells (no ASA and incubated in dark light (DL) for 10 min): (2) non-Rx cells that were exposed to ultraviolet light (UVL) for 10 min (UV effect); (3) cells treated with 1 mM of ASA (incubated in DL for 10 min; ASA effect); (4) cells treated with 0.5 mM of ASA and exposed to UVL 10 min (PDT effect); and (5) cells treated with 1 mM of ASA and exposed to UVL 10 min (PDT effect). In addition, negative controls were included, and where appropriate, the background readings they produced were subtracted for normalisation. These controls were: 1 mM ASA (with no cells) and 1 mM ASA with 10 min UVL (with no cells).

### 4.5. Survival Assay of Cryptococcal Cells

Following the handling of cryptococcal cells as per the above-mentioned experimental conditions, they were plated out. Specifically, the contents of wells were aspirated and transferred to corresponding 1.5 mL plastic tubes. A 1:10 serial dilution was made using sterile, distilled water. A 25 µL suspension of the diluted sample was dispensed to the centre of a corresponding YM agar plate. The suspension was then spread to create a lawn. The plates were then incubated for 48 h at 37 °C. At the end of the incubation period, colonies were counted to determine the survival rate of cells [56].

### 4.6. The Effects of PDT with ASA on the Cell Membrane and Cell Wall

It was expected that the ASA photolytic products would target cellular structures; therefore, determination of the effect of the resultant radicals on membrane integrity was then sought. To this end, the integrity of the cell membrane was assessed by measuring the amount of propidium iodide (PI) accumulated through damaged membranes. For the PI assay, the cells obtained from the following conditions: (1) non-Rx cells; (2) non-Rx cells and 10 min UVL (UV effect); (3) 1 mM-treated cells and 10 min DL (ASA effect); (4) 0.5 mM-treated cells and 10 min UVL (PDT effect); and (5) 1 mM-treated cells and 10 min UVL (PDT effect), were used in this experiment. The cells were then washed twice with PBS, and 99 μL of cells (from each experimental condition) were reacted with 1 μL of PI (1 mg/mL) in a black 96-well flat-bottom microtiter plate. The plate was immediately incubated in the dark for 30 min at 37 °C. The fluorescence was measured at 485 nm excitation and the corresponding emission at 538 nm using the Fluoroskan Ascent FL microplate reader [20].

#### 4.6.1. Effect of PDT on Cellular Outer Ultrastructure

Cells used for scanning electron microscopy (SEM) were obtained from: (1) non-Rx cells (no ASA and incubated in dark light (DL) for 10 min); (2) cells treated with 1 mM of ASA (incubated in DL for 10 min (ASA effect)); (3) non-Rx cells that were exposed to UVL for 10 min (UV effect); and (4) cells treated with 1 mM of ASA and exposed to UVL for 10 min (PDT effect).

All experimental cells were prepared for SEM according to the method of van Wyk and Wingfield [57]. Sodium-phosphate-buffered 3% glutardialdehyde and sodium-phosphate-buffered 3% osmium tetroxide were used to fix the cells before they were dehydrated in a graded series of ethanol solution. Next, the cells were critically point-dried, mounted, and coated with gold using an SEM coating system. Examination of cells was performed using a Shimadzu Superscan SSX 550 SEM. A number of images were taken from different positions on the stub. Furthermore, 100 randomly selected cells were considered, and their cell diameters were measured [57].

#### 4.6.2. Effect of PDT on the Expression of CAP64 Gene

The pellets obtained from the cells washed with PBS and number of cells were determined (5 × 10^5^ − 1 × 10^7^ CFU/mL). The cells were vortexed and centrifuged at 500× *g* for 5 min. Next, cells were aspirated, and the supernatant was discarded. A 700 µL GLT lysis buffer was added to the cells, which were mixed with beads. The cells were disrupted at 4 °C for 1 h using a Beadbug microtube homogeniser. The lysate was carefully removed from the glass beads and the total RNA was extracted following the manufacturer’s protocol. The nanodrop quantification to ensure the purity of the extracted total RNA was performed at wavelengths of A230, A260 and A280 nm, respectively. The RNA product was subjected to gel electrophoresis using 1.0% (*w*/*v*) agarose gel with 1X Tris-acetate-EDTA (TAE) buffer at 100 V for 35 min. The gel was then stained with 50 µL of ethidium bromide, and 100 bp DNA marker was used. The gel was visualised under a dark reader transilluminator. The associated DNA from the RNA preparations was removed by DNase. Next, the RNA samples were dissolved in RNase-free water. The real-time PCR reactions were performed in a total reaction of 25 µL containing 12.5 µL 2 × Rotor-Gene SYBR Green PCR Master Mix, 1.6 µL template DNA, 2.5 µL each of forward and reverse primers, 5.65 of RNase-free water, and 0.25 µL of Rotor-Gene RT Mix. The primers used for real-time PCR are listed in Table 1 and were designed for DNA sequences of actin and CAP genes. The RT-qPCR results of the *CAP64* gene were normalised to actin by carrying out the PCR under the same running conditions with an equal concentration of total RNA. Prior to PCR, reverse transcription was carried out. Reactions were incubated at 55 °C for 10 min. After reverse transcription, PCR was carried out and required an initial incubation step at 95 °C for 5 min. Two-step cycling was performed with 50 cycles. Each step comprised 2 steps: 95 °C for 5 s (denaturation step), and 60 °C for 10 s (annealing/extension step). The amplification was performed using a Rotor-Gene cycler. Melting curve analysis was performed after PCR completion to check the specificity of the reaction [58].

### 4.7. Effect of PDT with ASA on Cryptococcal Mitochondrial Membrane Potential (ΔΨM) and ROS Accumulation

Following the preparation of all cells (as mentioned in Section 4.4.), they were aspirated and dispensed into wells of a sterile black 96-well flat-bottom microtiter plate. The cells were then stained with 10 μL of the dye 5,5′,6,6′-tetrachloro-1,1′,3,3′-tetraethylbenzimidazolylcarbocyanine iodide (JC-1) dye, according to the manufacturer’s instructions. The plate was then incubated at 37 °C for 15 min. A Fluoroskan Ascent FL microplate reader was used to measure J aggregates (healthy cells) at excitation/emission wavelengths of 540/570 nm, while the monomeric forms (unhealthy cells) were measured at excitation/emission wavelengths of 485/535 nm [20].

In a separate experiment, ROS accumulation was also measured in a sterile black 96-well flat-bottom microtiter plate. A 90 μL suspension of the cells (treated as mentioned in Section 2.1.) was stained with 10 μL of the fluorescent 2′,7-dichlorofluorescein diacetate (DCFHDA; 1 mg/mL for 30 min in the dark at room temperature. Fluorescence was measured at excitation/emission wavelengths of 485/535 nm using a Fluoroskan Ascent FL microplate reader [20].

### 4.8. Effect of PDT Using ASA on the Health of Macrophages

The seeded macrophages (representing the standardised 1 × 10^6^ cells/mL) that were suspended in 100 μL of the media in a microtiter plate) were grouped into the following experimental conditions: (1) non-Rx cells; (2) non-Rx cells and UVL (UV effect); (3) 1 mM-treated cells and DL (ASA effect); (4) 0.5 mM-treated cells and UVL (PDT effect); and (5) 1 mM-treated cells and UVL (PDT effect). The plates were kept at room temperature and exposed to light (UV or dark) for 10 min. Following this, the media containing macrophages were aspirated and the cells were washed three times with PBS to remove excess ASA. To measure the metabolic activity of these cells, 54 μL of 2,3-bis (2-methoxy-4-nitro-5-sulfophenyl)-5-[(phenylamino)carbonyl]-2H-tetrazolium hydroxide (XTT; Sigma-Aldrich, South Africa) with 1 mM of menadione (Sigma-Aldrich, South Africa) were reacted with the cells. After three hours of incubation in the dark in a 5% CO_2_ incubator, which allowed the initiation of the tetrazolium reaction, the optical density (OD) was measured at 492 nm using a Biochrom spectrophotometer [59]. 

### 4.9. Statistical Analysis

For each study, three independent experiments were performed. No technical repeats were included for each independent experiment. Microsoft Excel was used to calculate mean values and the standard deviation of the means. The same programme was used to calculate the paired *t*-test to determine the statistical significance of non-Rx cells’ data, i.e., no ASA and incubated in dark light (DL) for 10 min, and the respective experimental condition being compared. For proper interpretation of the data, box plots were used, as recommended by Weissgerber et al., 2015 [60].

## 5. Conclusions

This old technology of light therapy has provided us with some evidence for combating cryptococcal cells under controlled laboratory conditions. It will be interesting to see if similar results can be obtained in a diseased animal with a cryptococcal skin infection; additionally, to determine if this treatment would be suitable for controlling facultative organisms, which can switch to anaerobic glycolytic fermentative pathways. Given the effect of PDT with ASA on the health of macrophages (which may be manipulated to disseminate cryptococcal cells), it would be prudent to now assess the impact of PDT with ASA on macrophage phagocytosis and to elucidate the molecular changes that may be enhanced to resolve internalised cells.

The application of this form of treatment against internal cryptococcal infections may, at the moment, be limited by the lack of appropriate technology to deliver it to affected organs. However, hand-held devices can be used to resolve cutaneous cryptococcal infections. Care should also be taken to avoid continuous photoreactions, given the harmful effects of UV light on the skin, particularly when subjects have concluded PDT administration and are exposed to the sun’s radiation. To this end, it is equally important to show the benefit of this treatment when administered to skin epithelial cells. Such a study could also include a molecule that could modify how skin epithelial cells receive UV radiation. Additionally, there are already some topical creams that have salicylates as an ingredient available on the market [27,49]. The latter demonstrates that there is scope to consider administering ASA as a photosensitiser to the skin.

## Figures and Tables

**Table 1:** Primer sequences.

Primer	Accession No.	Sequence (5’–3’)	Band Size
Actin	U10867	F-TGTACAATGGTATTGCCGACC R-CTGGTCCCTCAATCGTCCAC	200 bp
*CAP64*	L40026	F-GCCACGCCCACATTGACT R-ACTCTTCCTCGATCAATGTC	200 bp

## Data Availability

Raw data is available from the corresponding author upon request.

## Supplementary Materials

The following is available online at https://www.mdpi.com/article/10.3390/ph14050404/s1, **Figure S1:** Effect of PDT with ASA on the survival of *C. neoformans* H99 (**A**), *C. gattii* LMPE 053 (**B**) and *C. gattii* R265 (**C**). Non-Rx, non-treated cells; UVL, ultraviolet light; DL, dark light; CFU, colony-forming unit.
