# Supplementary figures and images for: The Repurposing of Acetylsalicylic Acid as a Photosensitiser to Inactivate the Growth of Cryptococcal Cells
Source: Pharmaceuticals (Basel). 2021 Apr 23;14(5):404. doi: 10.3390/ph14050404 (PMC8146328; doi:10.3390/ph14050404)

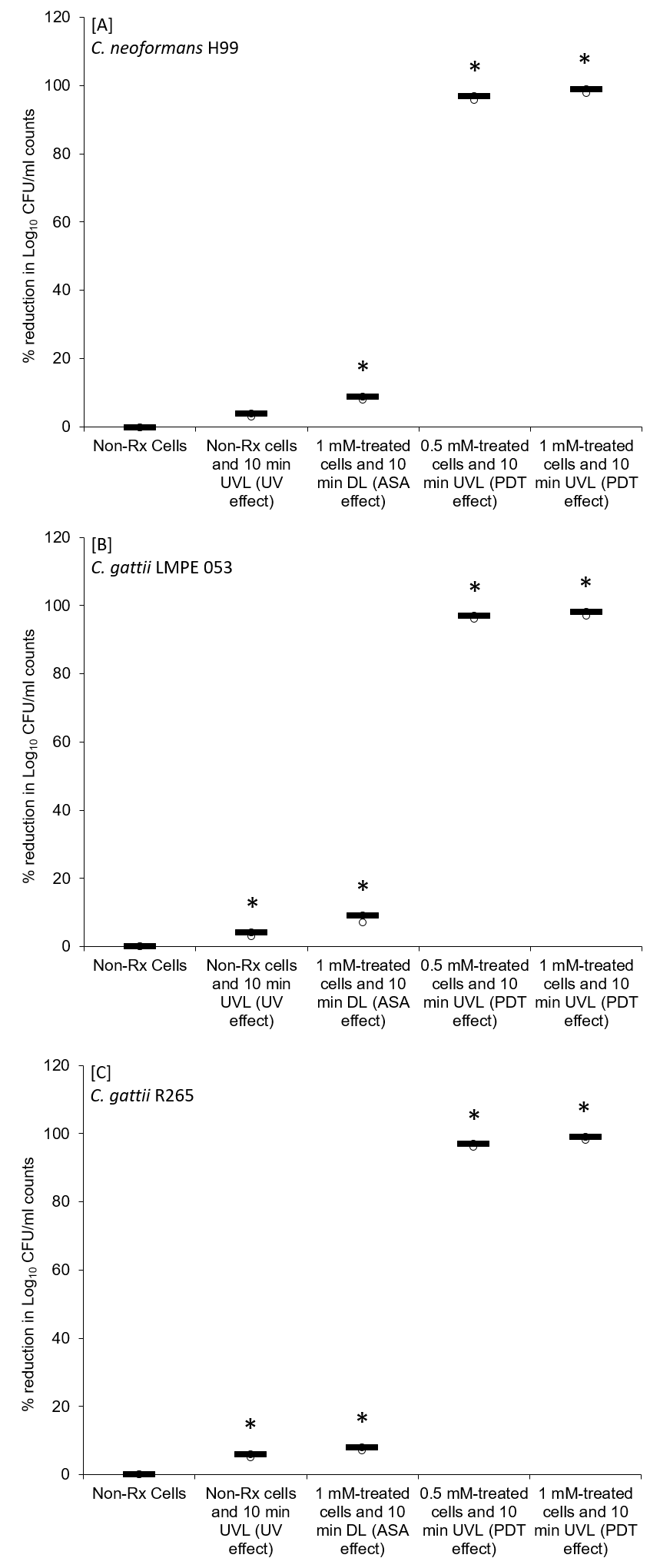

Supplement: Supplementary file 1 [file pharmaceuticals-14-00404-s001.zip › Fig S1.tif]
